# 

**Published:** 1886-05

**Authors:** 


### CONTENTS.

COMMUNICATIONS.

Oral Diseases........ 215
Abstract of a Lecture on Comparative Odontology........ 226
Bridge Work........ 231

PROCEEDINGS.

Summary of Discussions........ 243

SELECTIONS.

Dentition of Early American Races........ 252
No Right to Trifle with Human Life........ 255
Small-pox and Vaccination........ 255
Oil of Sassafras for Neuralgia........ 256
Choking........ 257
SALMAGUNDI........ 257

EDITORIAL.

To State and Local Dental Societies........ 262
American Medical Association........ 263
Obituary........264–5

